# Impaired mitochondrial respiration in human carotid plaque atherosclerosis: A potential role for Pink1 in vascular smooth muscle cell energetics

**DOI:** 10.1016/j.atherosclerosis.2017.11.009

**Published:** 2018-01

**Authors:** Craig K. Docherty, Andy Carswell, Elaine Friel, John R. Mercer

**Affiliations:** Institute of Cardiovascular and Medical Sciences, College of Medical Veterinary and Life Sciences, University Avenue, University of Glasgow, Glasgow, G12 8TA, Scotland, United Kingdom

**Keywords:** Atherosclerosis, Mitochondria, Oxidative phosphorylation, AMPK, Pink-1, Glycolysis, Metabolism, Vascular smooth muscle cells, p-VSMC, plaque vascular smooth muscle, OXPHOS, oxidative phosphorylation

## Abstract

**Background and aims:**

DNA damage and mitochondrial dysfunction are thought to play an essential role in ageing and the energetic decline of vascular smooth muscle cells (VSMCs) essential for maintaining plaque integrity. We aimed to better understand VSMCs and identify potentially useful compensatory pathways that could extend their lifespan. Moreover, we wanted to assess if defects in mitochondrial respiration exist in human atherosclerotic plaques and to identify the appropriate markers that may reflect a switch in VSMC energy metabolism.

**Methods:**

Human plaque tissue and cells were assessed for composition and evidence of DNA damage, repair capacity and mitochondrial dysfunction. Fresh plaque tissue was evaluated using high resolution oxygen respirometry to assess oxidative metabolism. Recruitment and processing of the mitochondrial regulator of autophagy Pink1 kinase was investigated in combination with transcriptional and protein markers associated with a potential switch to a more glycolytic metabolism.

**Results:**

Human VSMC have increased nuclear (nDNA) and mitochondrial (mtDNA) damage and reduced repair capacity. A subset of VSMCs within plaque cap had decreased oxidative phosphorylation and expression of Pink1 kinase. Plaque cells demonstrated increased glycolytic activity in response to loss of mitochondrial function. A potential compensatory glycolytic program may act as energetic switch via AMP kinase (AMPK) and hexokinase 2 (Hex2).

**Conclusions:**

We have identified a subset of plaque VSMCs required for plaque stability that have increased mitochondrial dysfunction and decreased oxidative phosphorylation. Pink1 kinase may initiate a cellular response to promote a compensatory glycolytic program associated with upregulation of AMPK and Hex2.

## Highlights

• Microdissection of human carotid plaques identifies defects in mitochondrial energetics.
• Identification and recruitment and processing of the mitophagy regulator Pink1 kinase.
• Compensatory changes associated with transcriptional and protein markers of glycolytic energetics.

## Graphical abstract

Schematic representation of the contribution of cardiovascular risk factors to the development of mitochondrial dysfunction, accelerated aging through Pink-1 dependent intermediates that control the balance between glycolytic and oxidative metabolism.

**Figure:**
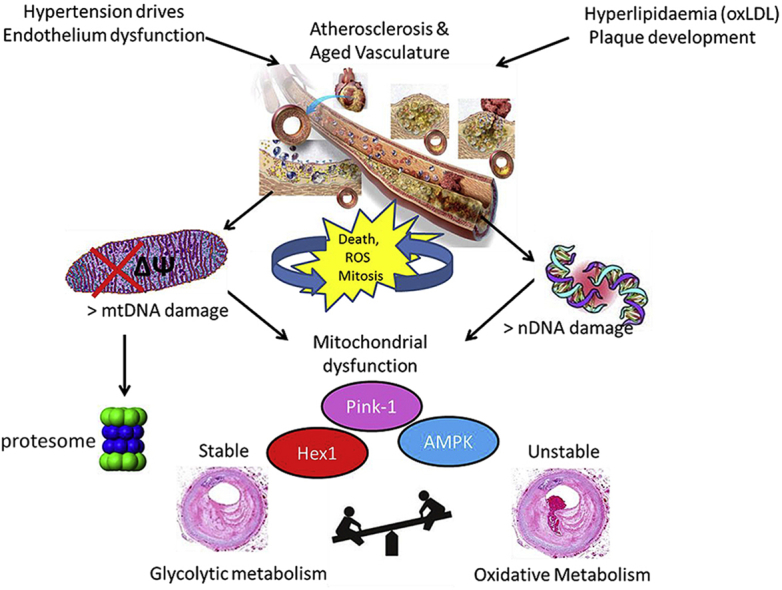

## 1 Introduction

Human atherosclerotic plaques are fibro-fatty arterial wall lesions which frequently rupture into the circulation and arguably are responsible for the majority of heart attacks and strokes. Risk factors such as inactivity, smoking and elevated cholesterol and lipid intake, generates a systemic inflammatory response that drives arterial wall and plaque inflammation [1]. Vascular smooth muscle cells (VSMC) are the main, synthetic and structural component of the vessel wall and plaque [2]. In response to injury during atherogenesis VSMC will transdifferentiate from contractile to a synthetic phenotype [3]. While VSMC's normally proliferate slowly in the plaque they also appear to have completed more cell divisions as evidenced by their decreased telomere length [4] which initiates a DNA damage response [5]. It is predicted that this need to proliferate and repair generates an ATP (adenosine triphosphate) demand and excess reactive species (RS) and oxygen free radicals that drives further DNA damage, genomic instability and mitochondrial dysfunction [6], [7], [8]. We predict that these characteristics accelerate atherosclerotic plaque formation and then limit VSMCs regenerative capacity [9], [10]. Indeed, additional radicals produced through vessel wall NADPH oxidase complexes also promotes endothelial dysfunction [11] and hypertension [12] which are known to initiate and exacerbate the progression of cardiovascular disease.

Mitochondria are the electrochemical batteries of the cell. Not only are they a source of ATP, they also buffer calcium beyond the traditional role of the endoplasmic reticulum (ER) and are important in initiating the intrinsic apoptosis cascade through caspase activation [13]. While the mitochondria retain a small 16 Kb genome, they lack the high fidelity repair systems of the nucleus. This property leaves the mitochondria vulnerable to the accumulation of damage which results in the loss of the proton motive gradient that drives the mitochondrial membrane potential (ΔΨm) required for ATP production and cell viability [14].

Pink-1 is the nuclear encoded protein kinase that under normal circumstances is constitutively synthesised in the cytoplasm and recruited to the outer mitochondrial membrane (OMM) where its mitochondrial targeting sequence (MTS) is cleaved. It is processed through the inner mitochondrial membrane (IMM) surface by the Presenilins-associated rhomboid-like protein (PARL) and passes through the mitochondrial outer membrane (TOM) complexes. In the presence of a mitochondrial membrane potential (ΔΨm), which is required for energy production, it is further processed through the translocases of the inner membrane (TIM) for retrograde transport out of the mitochondria [15] where it is then degraded. However, when ΔΨm is lost, Pink-1 becomes trapped at the outer mitochondrial membrane and flags the organelle as defective [16]. The ubiquitin ligase Parkin is recruited, binds Pink-1 [17] and orchestrates organelle recycling through the proteosomal degradation and engulfment [18]. This phenomenon of Pink-1 as mitochondrial biosensor is important and has not been investigated before in atherosclerotic plaques and VSMCs. If Pink-1 could also augment alternative energy pathways, than in the future pharmacologically switching VSMC energetics early in disease may offer a potential therapy.

We predict that during plaque formation VSMC mitochondria accumulate DNA damage leading to loss of ΔΨm. We predict Pink-1 will accumulate and become trapped at the outer mitochondrial membrane, resulting in oxidative energetic dysfunction. If Pink1 is present in human plaque VSMCs, it could be an important marker in detecting the failing cellular energetics of the plaque cap cells. This may allow recapitulation of the human plaque cell phenotype by transgenic manipulation of Pink-1 expression. An appropriate *in vivo* model could then function as a testbed for identifying alternative energy pathways and compounds that can activate energetic switching at earlier time-points during disease that could rescue *p*-VSMC phenotype.

To explore these ideas, we first investigated the energetic decline in plaque VSMC in response to nuclear and mitochondrial DNA damage and then the importance of Pink-1 in VSMC energy balance through potentially augmenting compensatory glycolytic pathways.

## 2 Materials and methods

### 2.1 Microdissection

Human carotid atherectomy specimens were obtained through the NHSCGC Biorepository and Pathology Services REC 10/S20704/60 after local ethical and patient approval. Each fresh sample was serially dissected, one piece used for histology, one for explant cell culture of wall VSMC nearest the plaque, one portion banked for RNA/DNA/protein extraction and the final portion used for oxygen respiration studies. For the respiration studies, the allotted piece was sub-divided into the wall; the VSMC rich residual adventitial and medial component of the artery, the core which contains the lipid rich inflammatory gruel, the shoulder which is rich in mitotic VSMCs and links the wall to the cap, and the cap, which is the often a thin overlaying VSMC sheet that maintains the plaque *in situ* and the site of rupture in the plaque. Plaques were imaged using Veho micro-capture v1.3. Primary VSMCs were cultured from explanted carotid specimens and identified by immunocytochemistry for α-smooth muscle cell actin (SMA) myosin heave chain (MHC) and Calponin as previously described [4], [19].

### 2.2 DNA damage assays

As previously described [20], DNA damage assays were performed in chamber-slides (Ibidi) cultured with primary explant VSMCs. Using immunofluorescence for detection, cells were fixed in ice cold methanol (10 min), blocked with 3% BSA/PBS, and probed with *p*-ATM^ser1981^ (red channel) and p-H2AX^ser 15^ (green channel) 1 h (Cell Signaling) and 30 min for fluorescent secondary antibodies (Cell signaling), using an isotype primary negative control and secondary. Total foci for both markers were scored per cell. Micronuclei were counterstained with biz-benzimide (blue channel) (Sigma). COMET assay was performed on slides in which isolated nuclei were derived from a cell alkaline lysis buffer and embedded in low melting point agarose with a 2% agarose support. Slides were electrophoresed at 1 V/cm for 10–20 min, then counterstained with ethidium bromide and scored. mtDNA PCR was performed as previously described [10].

### 2.3 Immunostaining assays

Pink-1 was assessed using an antiPink1 (Abcam 117091) raised to a synthetic peptide made to human PINK1 protein (residues 175–250). VSMC mitochondrial network was assessed by staining for the importer receptor-translocase of outer membrane (TOM20 - Ab56783). Cells were fixed in ice-cold methanol and blocked in 3% bovine serum albumin (BSA) and counterstained with Hoechst (H33342). (Zeiss, LSM 510). Mitopainter was added to visualise the coupled mitochondrial membrane potential, and uncoupling achieved through addition of CCCP (1–10 μM, Sigma). An IgG isotype and secondary antibody was used to control for non-specific binding.

### 2.4 Polargraphic whole tissue oxygen respirometry:O_2_K

Fresh biopsies of human tissues were obtained under local ethical approval with written consent from patients undergoing carotid endarterectomy procedures. Briefly, tissue was washed in BIOPS pH 7.1 and permeabilised with saponin (5 mg/ml). Samples were further washed 3 × 5 min in BIOPS before paired tissue samples (∼10 mg w/w) were added to the respirometry chambers (Oroboros Instruments, Innsbruck). Complex 1 respiration was stimulated with glutamate and malate and ADP before inhibition with rotenone to reveal a complex 1 dependent oxygen consumption rate (OCR). Stimulation of complex 2 dependent respiration was made by addition of the complex 2 substrate succinate and then inhibited by antimycin. Complex 4 respiration was stimulated with the electron donor ascorbate and artificial complex 4 substrate TMPD (N,N,N′,N′-Tetramethyl-*p*-phenylenediaminedihydrochloride). A final addition of excess cytochrome C showed no further increase in respiration, and confirmed the integrity of the respiratory chain preparation. All rates were normalised to dry tissue mass and immunohistology confirmed cell type and cell density for each region by eosin and haematoxylin (H&E) staining.

### 2.5 RNA extraction and real-time qPCR

Carotid arteries were selected based on their *en face* properties from our biobank of 40 samples (age range 50–76 years, with both sexes represented). Specimens were dissected into four areas: cap, core, shoulder and wall. Each area was finely minced using a sterile scalpel and placed into QIAzol solution. Stainless steel beads were added and samples were lysed using a TissueLyser ^(^™^)^ (Qiagen). Proteinase K (final concentration 100 μg/ml) was added to the QIAzol solution and incubated at 56 °C for 1 h. Samples were then placed back into the tissue lyser to fully disrupt the remaining tissue fragments. RNA species were then extracted with miRNeasy^®^ mini kit (Qiagen), quantified using a nanodrop spectrophotometer and normalised. RNA was then reversetranscribed to cDNA using the TaqMan^®^ reverse transcription kit (Applied Biosystems). Real-time PCR was performed using the Applied Biosystems 7900 HT real-time PCR system, following manufacturer's instructions. Specific TaqMan^®^ primer-probes were purchased from Life-technologies (Supplementary Table 1).

### 2.6 Microscopy

Bright field, confocal and time-lapse microscopy was undertaken as previously described, [20].

### 2.7 Western blotting

Carotid artery smooth muscle cells grown from carotid artery wall explants were grown in 6-well plates. 80–90% confluent cells were washed in PBS and lysed in 1% Lauryl maltoside detergent (Abcam)/PBS plus protease inhibitor cocktail (Sigma), scraped and sonicated. Twenty μg of protein lysates was fractionated on 4–12% gradient polyacrylamide gels and transferred to nitrocellulose membranes (Amersham). Membranes were then blocked in 1:1 Seablock (Thermofisher) and 1:1 Tris-buffered saline plus tween 20 (TBS/T). Membranes were incubated overnight at 4 °C with relevant primary antibody (Supplementary Table 2). Membranes were then washed in TBS/T and incubated with fluorescent secondary antibody (Life technologies; 1:15000) for 2 h. Membranes were then transferred to the LI-COR Odyssey infrared imaging system for visualisation. Densitometric analysis was then performed on LI-COR image studio light (version 5.2).

### 2.8 Glucose uptake assay

A luciferase based 2 deoxyglucose assay was performed using human plaque VSMCs (Promega J1341). Briefly, cells were stimulated and quiesced in a zero glucose media. 2-deoxy glucose (1 mM) was pulse incubated with the cultures using a 96-well plate format for ∼10 min. Cells were then lysed and neutralized before a custom lucigenic reaction mixture was added. Luminescence was then recorded using a 0.3 integration interval on a VICTOR, Perkin Elmer multi-label plate reader.

### 2.9 XF24 extracellular flux analysis

As previously described [20], 000 cells were plated into a Seahorse 24-well culture plate 24 h prior to the assay. These were then washed and quiesced with Seahorse low glucose buffered media. Using a 2-2-2 min (Mix-Wait-Measure) protocol, a pre-calibrated sensor plate was preloaded with 100 μl of Oligomycin (1 μM) port A, CCCP (1 μM) Port B and Rotenone and antimycin (1 μM each) Port C & D. The plate was pre-incubated and warmed at 37 °C. At the end of the assay, reference wells were taken for protein determination and equal cell loading.

### 2.10 Histology

Histology was performed as previously described [19], [20], [21], [10]. Goat anti-rabbit HRP secondary antibodies (A6154, Sigma) and Vector labs DAB secondary HRP staining kit (SK4100) were used for staining, including a secondary negative control and an infiltrating breast ductal carcinoma used as positive control (AMSBIO ™ CU2005/17).

### 2.11 Statistical analysis

All data are presented as mean ± standard error of the mean (SEM), where appropriate. *p* values were calculated using Student's unpaired *t*-tests (two-tailed distribution). Statistical significance was considered as *p* < 0.05 (one star), *p* < 0.01 (two stars) or *p* < 0.001 (three stars). All analysis was performed on Microsoft Office Excel 2007 or Graph pad Prism (v 5). All graphs were produced on MS Excel or Graph pad Prism (v 5). One-way analysis of variance (ANOVA) was used to determine significant difference between two or more independent groups.

## 3 Results

### 3.1 Human plaque microdissection and phenotyping

**Fig. 1:**
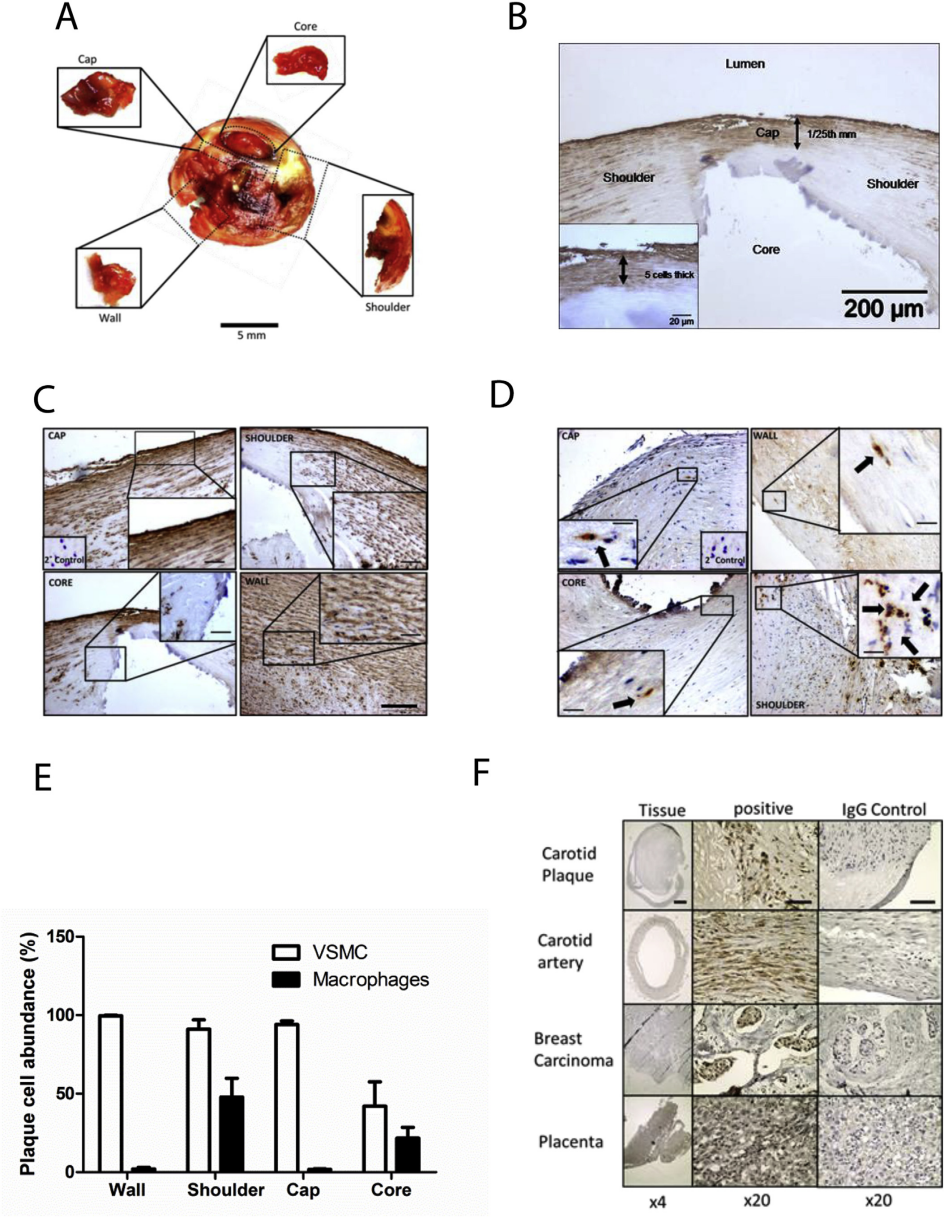
Human carotid plaque composition; VSMC and macrophage regional abundance. (A) Carotid plaque cross section; thin fibrous cap, core removed *en bloc*; adventitial wall dissected away from overlying structures with the shoulder region a mixture of wall, core and cap. (B) Plaque histology for smooth muscle stained (a-SMA) carotid plaque, gross morphology identifying each of the regions of interest: (i) wall, (ii) shoulder, (iii) cap, (iv) core. (C) α-SMA + VSMCs. (D) Mac-3 + monocyte/macrophages (scale bar 100 μm). (E) Cell abundance in plaque regions (n = 7) and results quantified. (F) Histology of Pink expression (High power 20X, low power 4X).

To determine the role of mitochondria in regulating the energetic phenotype of the plaque, we generated a biobank of human carotid endarterectomy plaque samples. From 60 samples, we micro-dissected those plaques with the *en face* properties that clearly showed the four identifiable regions of cap, core, shoulder and wall (Fig. 1A). Cross sections of plaque tissue were immuno-stained and scored for cell type and density (Fig. 1B). In particular, for the two most important types that determine plaque fate: VSMC and monocyte/macrophages. A refinement of previous work showed the plaque cap likely had a similar cell density to the wall, with the majority of these cells being either smooth muscle actin (SMA+) (Fig. 1C) and/or Myosin Heavy Chain (MHC+) (Supplementary Fig. 1) positive cells. Less than 2% of cells being infiltrating immune cells, such as mac3+ monocyte/macrophages (Fig. 1D). In contrast, the plaque shoulder region appeared as the most dynamic region, with almost twice the density cells and a much higher proportion of infiltrating macrophages, which was quantified (Fig. 1E) and tabulated (Table 1). While the largely acellular lipid core had the lowest cell density, the plaque shoulder had the highest one, with the combined scoring in some regions suggesting greater than 100% of marked cells, possibly from antigenic debris left over from apoptosis and necrosis. To assess plaques for markers of mitochondrial dysfunction where oxidative metabolism was compromised, we immuno-stained for Pink-1 kinase. Pink-1 expression was increased in the plaque cap region *vs*. the control. (Fig. 1F).

**Table 1:** Compositional analysis of intra-atherosclerotic plaque from human carotid endarterectomy specimens.

	Wall	Shoulder	Cap	Core
Cell density 1.0xE5/μM^2^	81.9 ± 6.2	146 ± 20.7	91.3 ± 13.2	32.8 ± 6.4
VSMC (%)	99.4 ± 0.5	90.9 ± 5.0	94.1 ± 1.9	41.9 ± 11.2
Macrophages (%)	1.9 ± 0.6	48.0 ± 9.8	1.6 ± 0.3	21.7 ± 4.9

### 3.2 DNA damage and mitochondrial dysfunction in plaques

**Fig. 2:**
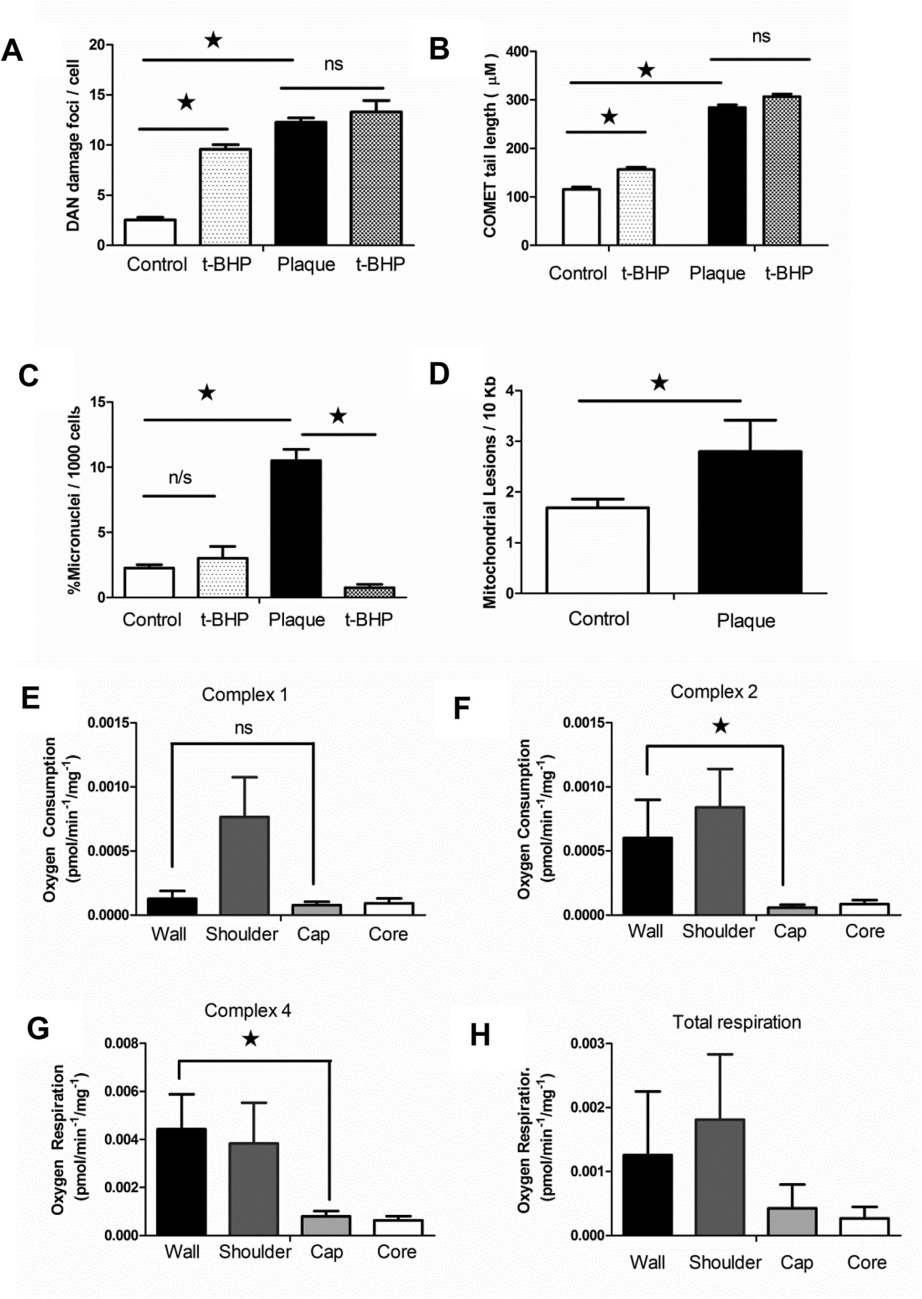
Human carotid plaque tissues have increased nuclear and mitochondrial DNA damage and defects in oxidative phosphorylation. (A) DNA damage foci from control VSMC plaque cells ± hydrogen peroxide (t-BHP) treatment (n = 4). (B) Nuclear repair assessed through COMET assay for control and plaque VSMC ± t-BHP (n = 4). (C) Nuclear DNA micronuclei in control and plaque VSMC ± t-BHP (n = 4). (D) Mitochondrial DNA damage in control and plaque VSMC (n = 7). (E) Total plaque tissue oxygen respiration for key regions (n = 7). (F) Tissue oxygen consumption rate (OCR) for Complex 1 dependent respiration (n = 7). (G) Tissue oxygen consumption rate for Complex 2 dependent respiration (n = 7). (H) Tissue oxygen consumption rate (OCR) for Complex 4 dependent respiration (n = 7) (non-binomial distribution tested by Mann Whitney *U*-test *p = 0.05).

From our human carotid plaques tissue samples, we generated explanted primary human VSMCs using fresh medial portions derived from patient's undergoing carotid atherectomy (approved NHS GC&C Biorepository application 107 REC 10/S20704/60). These primary cell lines were also routinely immuno-stained and confirmed as expressing α-SMA and myosin heavy chain (Supplementary Fig. 1), and additional VSMC markers, including vimentin and calponin. We then immuno-stained the derived cells for their nuclear DNA damage foci, combing expression of *p*-ATM and p-H2AX foci/cell (Cell signaling, UK) in the presence and absence of the hydrogen peroxide analogue, t-BHP (Sigma, UK) (Fig. 2A). In addition, we scored their DNA repair capacity (Fig. 2B) using the COMET assay (Comet Assay IV™) and unrepaired DNA as extruded as micronuclei (Fig. 2C). Using a semi-quantitative PCR method that compared amplification efficiency between a large 10 Kb and a small 250 bp sequence of the mitochondrial genome, the number of lesions 10 Kb was determined (Fig. 2D). To assess the functional impact of this across the plaque, fresh sub-plaque regions were micro-dissected and assessed for oxygen consumption and the total contribution of each respiratory complex was determined. Respiration was consistently decreased across the plaque when compared to the wall region (Fig. 2E–H). In particular, complexes 2 and 4 respiration were significantly compromised (Fig. 2G and H).

### 3.3 Pink-1 flags dysfunction plaque cap tissue and cells

**Fig. 3:**
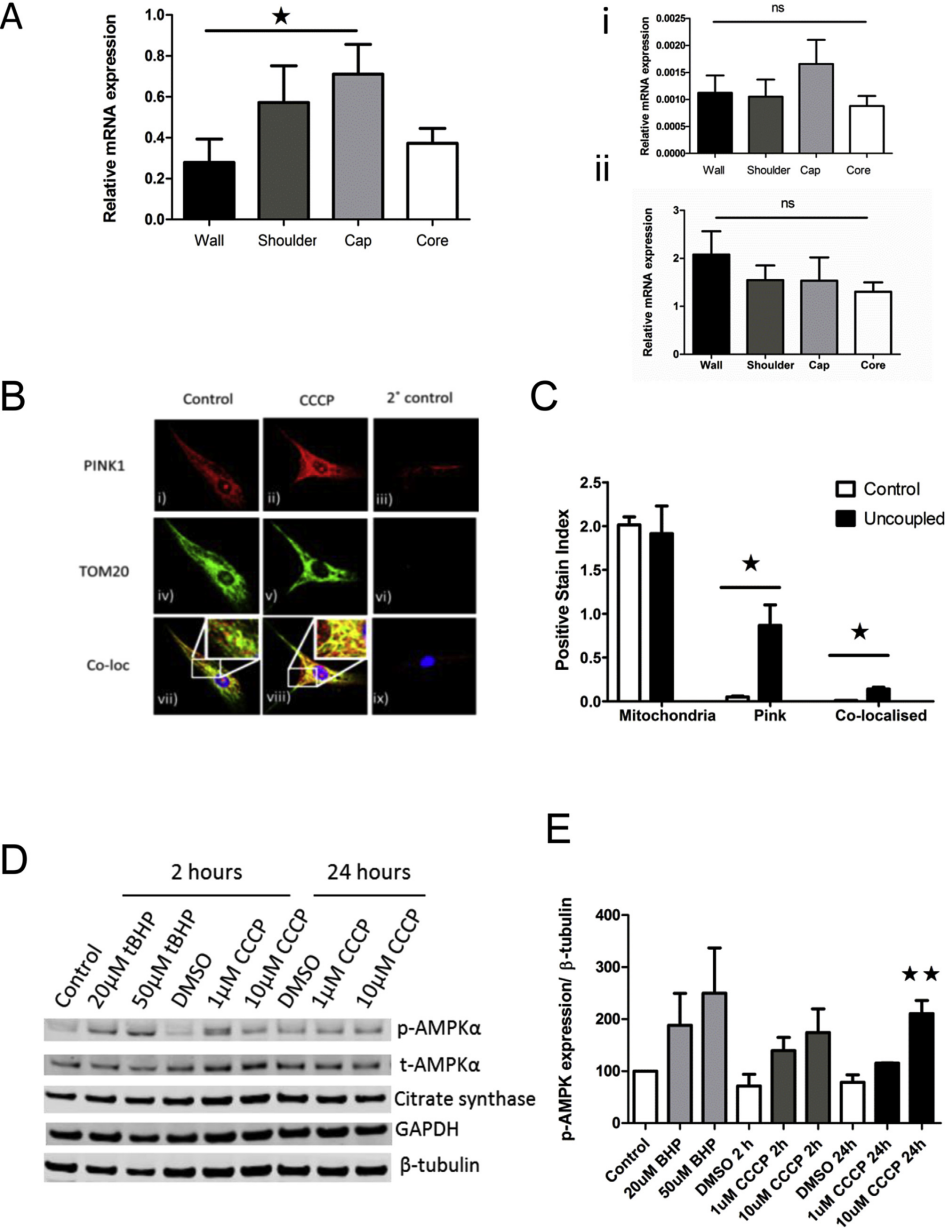
Mitochondria abundance and Pink1 expression in human plaque cells and tissues. (A) *Pink-1* mRNA expression in isolated plaque tissue using real-time Taqman ΔΔCt, normalised to citrate synthase, (i) citrate synthase mRNA expression (n = 5), (ii) mitofusin expression (n = 5). (B) Confocal immunocytochemistry of Pink1 protein in isolated plaque cells after uncoupling. (i) Anti Pink1 Ab mitochondrial staining. (ii) Network changes after uncoupling with CCCP (1 μM). (iii) Secondary only control. (iv) Anti-TOM20 organelle staining. (v) Anti-TOM20 after uncoupling. (vi) Secondary IgG antibody control, (vii) with localisation, (viii) and after uncoupling. (C) Quantification of Pink1 and Tom20 localisation after uncoupling using the non-potentiometric dye (mitopainter) and Pink-1 ± uncoupling with CCCP. (D) Western blot of phospho and total AMPK abundance and before and after uncoupling with CCCP and the DNA damaging agent t-BHP (n = 3) normalised to beta tubulin. (E) Quantification of *p*-AMPK protein expression. (ANOVA * *p* = 0.05).

To determine the mechanism by which oxidative phosphorylation may be compromised, we assessed the abundance of key mRNA transcripts associated with mitochondrial health in VSMCs. We found a significant upregulation of Pink 1 kinase mRNA (Fig. 3A), corroborating our finding in plaque tissue. No difference in plaque VSMC mitochondrial content was detected when using the mRNA expression of the nuclear encoded Krebs cycle enzyme citrate synthase (Fig. 3A–i) or the mRNA expression of the mitophagy regulator MFN2 (Fig. 3A–ii). The subcellular VSMC localisation of Pink1 protein relative to a mitochondrial protein marker TOM20 was determined using confocal microscopy (Fig. 3B). After uncoupling ΔΨm with the respiratory chain uncoupler CCCP (1 μM), Pink1 levels stabilised and were co-localised with TOM20 on the mitochondrial membrane, observed as a shift from green (vii) to yellow staining (viii), and this effect could also be quantified using the non-potentiometric dye mitopainter™ (Fig. 3C).

Loss of oxidative metabolism is predicted to decrease abundance of the adenosine triphosphate (ATP) and increase the energy sensor AMP kinase (AMPK). To assess this aspect, isolated VSMCs were stimulated with either hydrogen peroxide (t-BHP) or uncoupler (CCCP), to mimic mitochondria dysfunction. Western blotting assessed total and phospho-AMPK and phospho-Pink1 protein expression during mitochondria recruitment, processing and activation. While hydrogen peroxide could increase total Pink1 protein (65 KDa) at both low and high concentrations, the active 55 kDa Pink1 accumulated only after uncoupling with CCCP. This confirmed the uncoupling as essential for Pink-1 activation in VSMCs, associated with a concomitant increase in the energy sensor AMPK (Fig. 3D). This effect was greatest after 24 h stimulation and was quantified (Fig. 3E), and was also observed after incubation with a vascular relevant stressor, oxidised LDL (Supplementary Figs. 1E and F).

### 3.4 Plaque transcript analysis

**Fig. 4:**
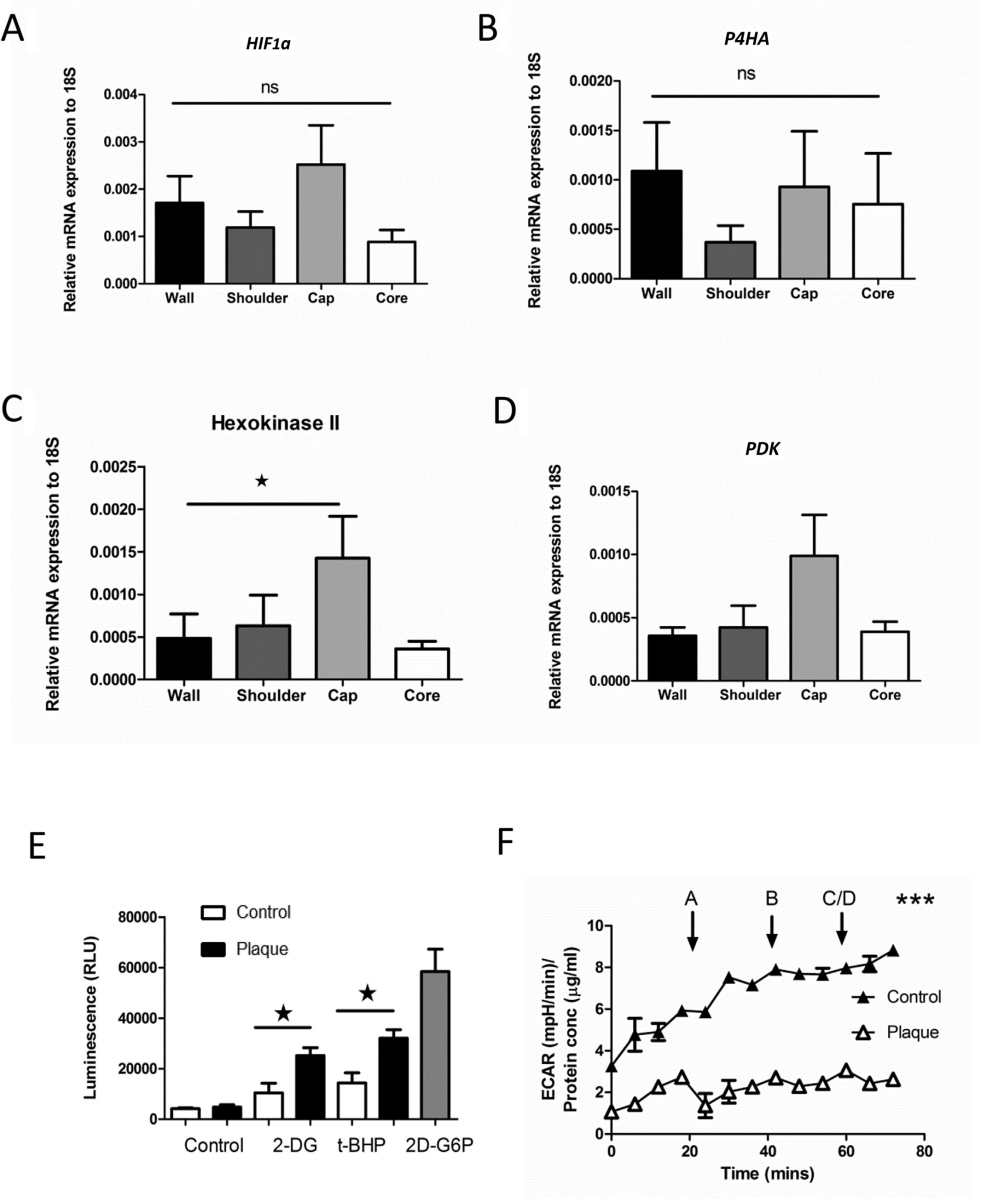
mRNA expression of regional plaque markers and glucose uptake in human plaque cells. (A) Expression of plaque *HIF-1*α /18S ΔCt change in expression. (B) HIF-1α degrading prolyl hydroxylases (*PHD*)/18S. (C) Hexokinase II /18S expression. (D) Pyruvate dehydrogenase kinase /18S (n = 4–6). (E) Glucose uptake in aortic control and carotid plaque derived cells (n = 2) (ANOVA * *p* = 0.05). (F) Seahorse XF24 extracellular acidification rate (ECAR) of control and plaque VSMC during uncoupling with CCCP (1 μM). Student *t*-test (** *p = 0.001) (n = 3).

Interacting factors that could augment Pink1 were explored using MetaCore™ network analysis. This identified a number of potential proteins; using real-time qPCR expression analysis, we validated potential targets. We found no changes in the constitutive expression of either HIF-1alpha or its regulatory prolyl hydroxylase P4H (PHD1) (Fig. 4A and B).

**Fig. 5:**
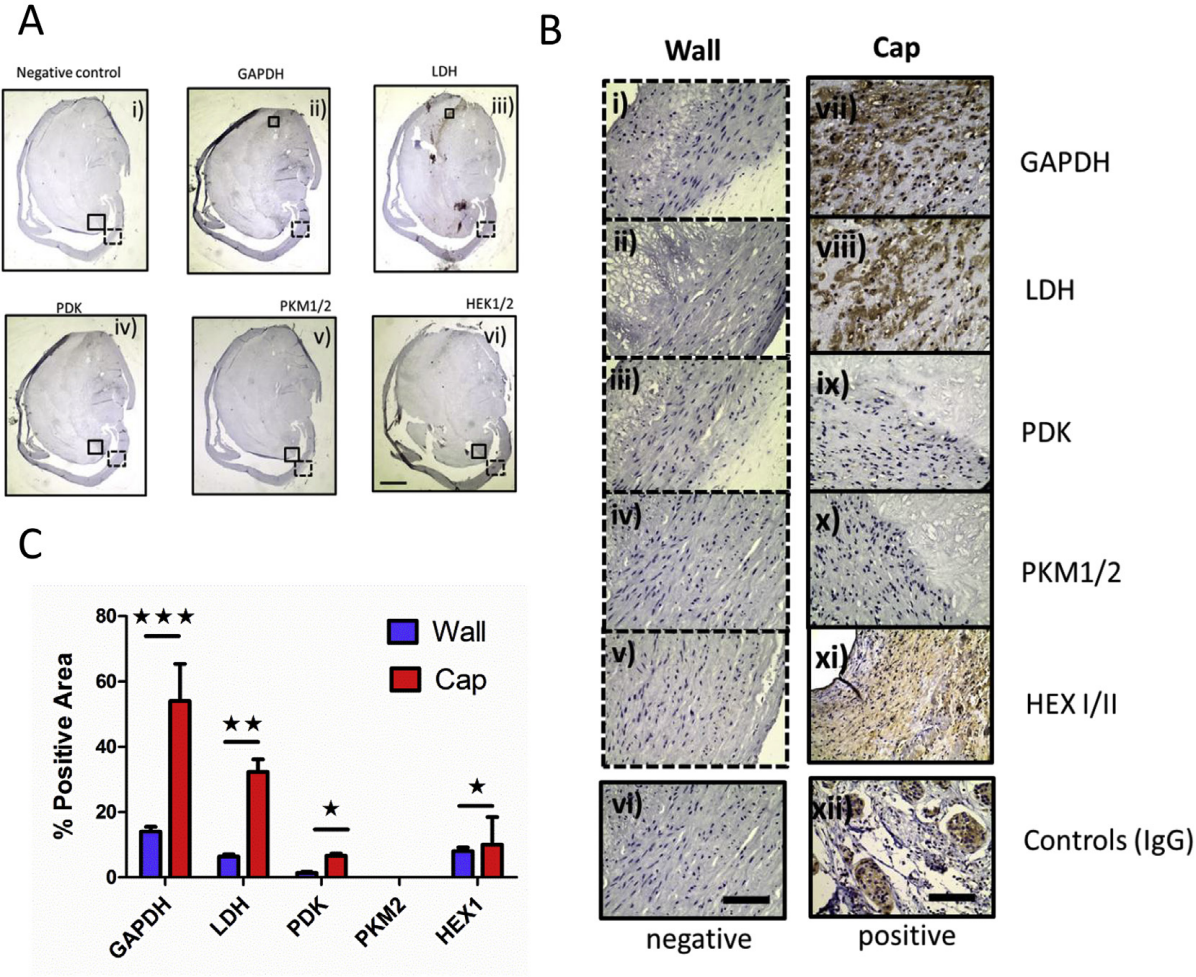
Comparison of glycolytic markers between carotid plaque wall and cap. (A) Human carotid plaques were compared across whole plaque sections (low power 40× scale 500 μm) (i-vi). (B) High power comparisons made: (i) glyceraldehyde 3-phosphate dehydrogenase (GAPDH), (ii) lactate dehydrogenase (LDH), (iii) pyruvate dehydrogenase kinase (PDK), (iv) pyruvate kinase muscle (PKM) isoenzyme 1/2, and (vi) hexokinase (HEXI/II) (n = 4). Including irrelevant IgG isotype and secondary negative control and a tumour positive control (AMSBIO) 400× (scale 100 μm). (C) Quantification histogram of glycolytic markers excluding nominal PDH expression, comparing between plaque cap and wall (n = 3) (2 way ANOVA* *p* = 0.05, **p = 0.01, ***p = 0.001).

We did identify elevated hexokinase II transcripts in the cap, the first enzyme of the glycolytic pathway (Fig. 4C). In addition, pyruvate dehydrogenase kinase (PDK) the regulator of PDH activity, the enzyme responsible for converting pyruvate to acetyl CoA, was also elevated, however, it was not statistically significant (Fig. 4D). To test if plaque-derived cells had an altered capacity to increase glycolysis, we measured glucose uptake using a non-radioactive bioluminescent method based on uptake of 2-deoxyglucose-6-phosphate (2DG6P) (Promega-Glucose UptakeGlo). We found plaque-derived cells had increased uptake in the presence of absence of the DNA damaging agent t-BHP (Fig. 4E). We further tested the ability of plaque cells to maintain glycolysis during respiratory uncoupling using the Seahorse XF24 bioanalyser. The XF24 measures extracellular acidification caused by protons (H+ ions) generated during catabolism of glucose to lactate, but also the release of carbon dioxide during glucose oxidation. When comparing carotid plaque VSMC with control aortic VSMCs, we found a significant (*p* = 0.001) and sustained increase in ECAR (Fig. 4F). To validate these changes, we also assessed human plaque tissue for their protein abundance for glycolytic and TCA proteins such as PDK (Fig. 5). The abundance of GAPDH, LDH, PDK and Hex was all significantly increased in the plaque cap compared to the wall (Fig. 5A and B) and levels were further quantified (Fig. 5C). To try and understand the implications of these changes in the plaque, we assessed isolated plaque cells response to genotoxic stress. Using time-lapse microscopy, over 48 h, we found plaque cells proliferated more slowly and underwent apoptosis more frequently, reflecting the terminal nature of these explanted cells (Supplementary Fig. 2).

## 4 Discussion

The majority of cardiovascular related deaths are attributable to atherosclerotic plaque rupture [22]. The thinning of the overlying *p*-VSMC cap precipitates rupture and erosion and is likely responsible for the majority of cardiovascular related-mortality from myocardial infarctions and strokes.

The phenotype of the vascular smooth muscle cells that constitutes the vessel wall and plaque has continued to be evaluated over many years ([23], [24], [25]). Our previous work has investigated the DNA damage response using transgenic models, but the physiological and energetic responses of these cells in human diseased samples remains to be explored. We now provide the implications of this phenotype in human carotid plaques and a rationale for the metabolic decline of plaque cells.

We identified the plaque cap cells in terminal decline, as tentatively having an alternative bioenergetic profile compared to the vessel wall and neighbouring plaque structures. We have shown that when co-stained with markers of VSMC content, the cap likely contains no significant difference in VSMC content compared to the wall, although some “vulnerable regions” clearly have fewer cells. More importantly, there appears to be a significant drop in cap tissue oxygen consumption, in particular during Complex II and IV dependent respiration. Assessment of mitochondrial abundance identified normal plaque cap mitochondrial quantity but at a reduced quality, and this was linked to the recruitment and retention of Pink1. This process may promote induction of *p*-AMPK as an energy sensor and induction of glycolytic enzymes such as hexokinase 2 and mitochondrial PDH. PDH is important as it regulates the metabolic flexibility between glycolysis and oxidative metabolism. The implication of these data was supported by an increased uptake of the glucose analogue 2-deoxy-glucose in isolated plaque cells basally and after stress (t-BHP). Moreover, plaque-derived VSMCs when assessed for glycolytic flux, compared to aortic controls, were also more glycolytic during uncoupling. Increasing glycolysis would be predicted to restore ATP abundance, at least when assessed in isolated cells. We predict this could be a survival response to the energetic failure in the plaque cells and, if invoked earlier in disease, could be a therapeutic strategy.

Our ability to demark energetic changes geographically within the plaque sheds new light on how plaque tissue may respond during development and rupture. Understanding how these changes manifest as alterations in VSMC mitochondrial respiratory chain complex activities, in particular Complex II and Complex IV dependent respiration, is new. This work highlights the heterogeneity of regional cellular respiration within the plaque and how plaque VSMCs respond to genotoxic stress. Further work is now required to understand the temporal energetic demise of plaque mitochondria and further substantiates the presence of a tentative alternative energetic program in plaque VSMCs. If this program is initiated by Pink1 kinase and dependent on hexokinase I/II, it could be a useful starting point to intervene in these processes. Indeed, in our cellular model, we found the energy sensor AMPK was also upregulated in response to uncoupling and DNA damage, suggesting the loss of ATP (when ADP/AMP is high) may be sufficient to trigger AMPK to restore glycolysis.

We report an almost 3-fold elevation in hexokinase II expression in plaque cap specimens. Hexokinase II (Hex II) is the most abundant isoform of the hexokinase family [26], and its elevated intracellular activity is a crucial step in glycolysis, through phosphorylation of glucose to glucose-6-phosphate [27]. Its abundance has been positively correlated in plaque carotid specimens with concomitant elevation of glucose transporters (Glut1-3) [28]. Hexokinase II is the only family member to associate with the outer mitochondrial membrane and has previously been identified after a genome-wide screen of small hairpin RNA, reported as critical for the Pink1 dependent ubiquitin ligase Parkin to be recruited [29].

Hex II expression in the plaque cap may potentially drive the proposed energetic switch through driving elevation of PDH activity and block the pyruvate dehydrogenase complex (PDC) activity required for oxidative metabolism, thereby favouring glycolysis in response to mitochondrial dysfunction [30], [31], [20]. Note, that while hexokinase I is the predominate isoform in humans, hexokinase II has a greater than 70% homology, but is usually found in lower abundance.

These new insights provide a rationale to interrogate Pink1 binding partners in atherosclerosis and to discover if plaque VSMCs can energetically switch from oxidative phosphorylation to glycolysis earlier in atherogenesis. Pink-1 ablation could be used to model the features of energetic dysfunction and develop the implications of energetic switching further. Characterising these changes during disease could be undertaken with a return to appropriate inducible and lineage specific transgenic models of Pink1 ablation.

## Conflict of interest

The authors declared they do not have anything to disclose regarding conflict of interest with respect to this manuscript.

## Financial support

This work was supported by the University of Glasgow: Strategic fund 252-146123-001. This work was support by the British Heart Foundation centre of excellence research award RE/13/5/30177.

## Author contributions

Dr Docherty performed the Taqman, Western blotting and immunocytochemistry work.

Mr Carswell performed the histology, Mrs Friel generated and banked the carotid endarterectomy specimens and human cells lines. Dr Mercer performed the oxygen respirometry, energetics data and drafted the manuscript and envisioned the project.
