# Supplementary material for: Impaired mitochondrial respiration in human carotid plaque atherosclerosis: A potential role for Pink1 in vascular smooth muscle cell energetics
Source: Atherosclerosis. 2018 Jan;268:1–11. doi: 10.1016/j.atherosclerosis.2017.11.009 (PMC6565844; doi:10.1016/j.atherosclerosis.2017.11.009)
Supplement: Online data [file mmc1.docx]

**Supplemental Figure Legends**

**Supplemental Figure 1)** Mysoin heavy chain plaque staining and evidence of senescence in human plaque tissue and cells. **(A)** Myosin Heavy Chain (MHC) expression with i) IgG secondary antibody control, ii) Isotype primary antibody control (low x4 and high power x60) scale bar 500μm. **(B)** Human atherosclerotic plaque dual stained for expression of α-SMA and SAβG (x4) and **(insert)** high power of positive cells in the plaque as arrowed (x60) scale bar 50μm. **(D)** Quantification of the accumulation of plaque cell SAβG in culture with increased passage (n=3). Induction of Pink 1 by oxLDL - **(E)** incubation of VSMC with fluorescently labelled oxidised LDL Uptake and sequestration observed as yellow staining arrowed. **(F)** Pink 1 protein expression in VSMC isolates incubated with oxLDL and normalised to beta-actin (n=2).

**Supplemental Figure 2) – Proliferation and apoptosis in plaque cells.**  Cumulative cell proliferation and apoptosis in control and stimulated, t-BHP (10μM) plaque VSMCs over 48 hrs by time-lapse (n=5) (students t-test * *p=*0.05).
