# Supplementary figures and images for: Impaired mitochondrial respiration in human carotid plaque atherosclerosis: A potential role for Pink1 in vascular smooth muscle cell energetics
Source: Atherosclerosis. 2018 Jan;268:1–11. doi: 10.1016/j.atherosclerosis.2017.11.009 (PMC6565844; doi:10.1016/j.atherosclerosis.2017.11.009)

Supplemental Figure 1

**A)**

**B)**

**C)**

**D)**


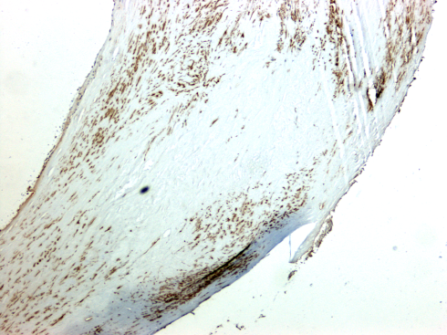

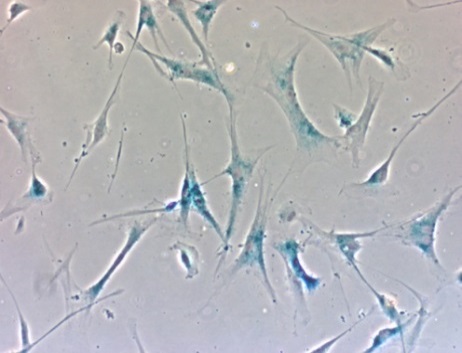

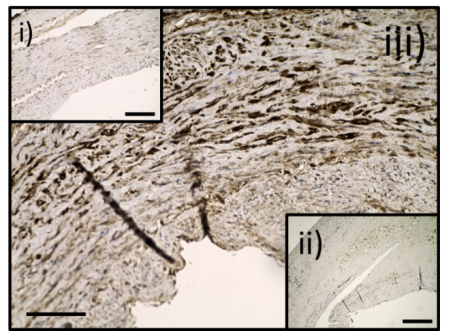

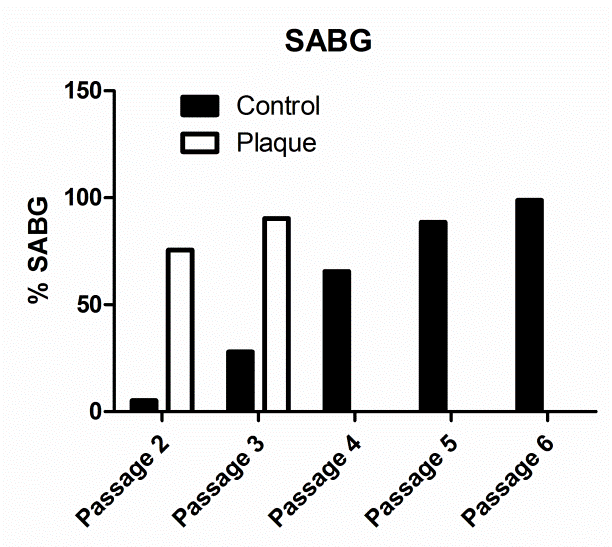

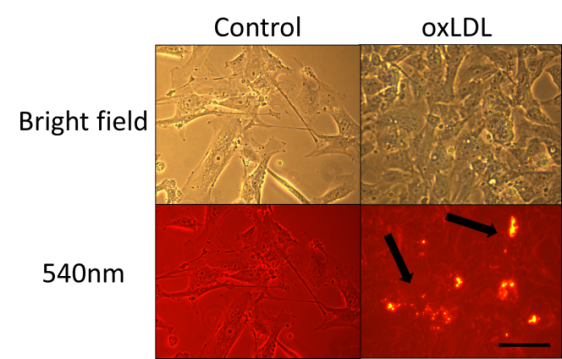


**E)**

**F)**


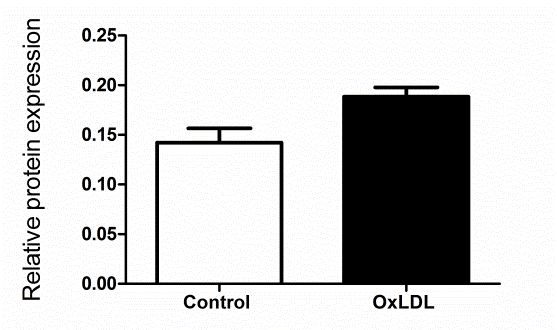

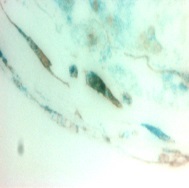

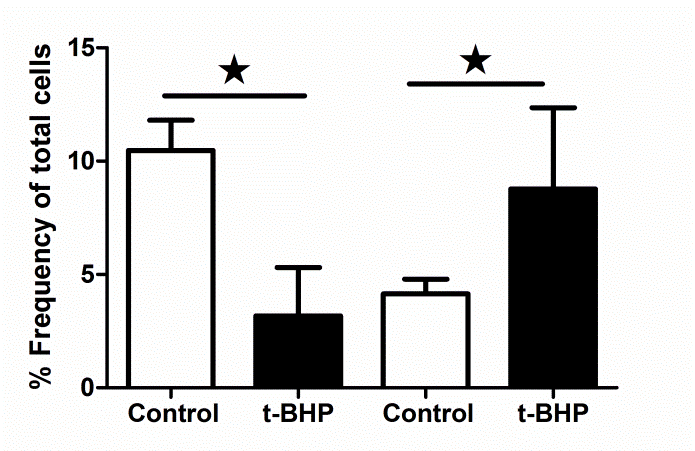

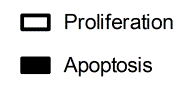


Supplemental Figure 2

Supplement: Online data [file mmc2.docx]
